# Application of Gymnastics to the Treatment of Disease

**Published:** 1824-03

**Authors:** P. H. Clias


### ART. VI.—*Application of Gymnastics to the Treatment of Disease.* By P. H. CLIAS, Esq.

MISS A. B., aged sixteen years, had been affected for several years with a curvature of the six upper dorsal vertebræ, from the right to the left side; during which period her condition was as follows:—A general weakness, particularly of the respiratory organs; she was extremely pale and thin; her sleep much disturbed, and her appetite nearly gone; she had an obstinate cough; her voice was weak, and nearly inaudible; her menses suppressed. She had a continued pain in the left side, resulting from the pressure of stays, which she had worn for some months with the expectation of re-establishing the straight position of her back. Her head reclined upon her chest; and the false ribs of the left side were bent one over the other, and forced inwards. In this condition, the young lady was confided to my care on the 22d of October, 1822, by a physician of this town, who had seen and visited this lady for some years. I may be allowed to say that it was almost with repugnance that I engaged to employ my system of exercise in the case of a person who appeared to be nearly in a dying condition, but the entreaties of the relations, and the solicitations of the medical attendant, were so urgent, that I could not forbear making the trial. The table of exercises which I employed in this case will give a just idea, to those persons who interest themselves in this practice, how far I deserved the confidence reposed in me. If I have not given my reasons for adopting each particular exercise, it is because I thought the intelligent practitioner would easily understand their application in different cases.

### *Table of the Gymnastic Exercises.*

1. Prolonged inspirations, the patient sitting.
2. Prolonged inspirations, the patient standing, the arms fixed.
3. The same exercise, the arms hanging down.
4. The same, the arms extended horizontally.
5. The same, the arms fixed to a horizontal pole.

6. Deep inspiration, and counting a certain number without drawing the breath.
7. Movement of the feet on the ground, the patient sitting.
8. Deep inspiration, the patient lying on the left side, and leaning on the elbow.
9. In the same position, to raise and to lower the body.
10. Walking slowly, and making deep inspirations.
11. Walking a little faster, and counting several steps, without drawing breath.
12. Bending without rising, the weak hand fixed above.
13. Piaffer* with both hands fixed to the horizontal pole.
14. Bending the body, bearing a weight in the weak hand.
15. Piaffer, bearing the same weight in the weak hand.
16. Lifting up a small box from the ground with both hands.
17. The same exercise with the weak hand.
18. To declaim without moving.
19. The same, walking slowly.
26. Singing without drawing breath.

*Motion of the Arms, the Patient standing.*

21, 22, 23. Movements of balance simple, in front and on one side.
25, 26, 27. Develope other motions of the arms, difficult to describe without diagrams.
28. To imitate the motion of sawing, the patient placed below.
29. The same, the patient placed above.
30. The above exercises with the weak hand only.
31. To draw upon a spring with the weak hand only.
32. The same, the arms and body being fixed.
33. Seated on the ground, to rise with the assistance of the arms, the feet fixed.
34. Lying down horizontally, to raise the body without the assistance of the arms.

*Continuation of Exercises in the Room.*

35. Exercise of the arm with a pulley, the patient sitting, the body fixed.
36. The same with the weak hand only.
37. The same, the head fixed in a straight direction.
38. The same, direction of the head to the weak side.
39. Walking some distance, giving the weak arm to a tall person.
40. Lying on the chest, to raise the body backwards.
41. Sitting on the ground, to pull a stick, first with both hands, then with the weak one.
42. Piaffer, leaping up with a weight in the weak hand.
43. The cross step, in the same manner.
44, 45, 46. Other movements, not explainable without diagrams.

---

* Piaffer is a term in the manege, which can be, perhaps, best understood by the military phrase "marking time."

47. To rise and to fall, the knees fixed to the arms.

48. To bend the lower extremities, the arms fixed, the weak one higher than the other.

On the 27th of November, the cough having entirely ceased, and the progress of the patient giving me reason to expect the happiest results, I began to employ frictions during the exercise on the diseased parts. At this time I also took with her the first promenade, the weak arm supported, and afterwards in a carriage.

On the 12th of January, 1823, circumstances obliged me to quit my young patient. At this time she was so much recovered, that she could, without inconvenience, resume her lessons in singing, playing, and drawing, and walk several miles without fatigue. The cough and pain in the side had entirely ceased; she had an appearance of health, and her spirits were good. All the animal functions were perfectly restored.

